# Availability and price of fruits and vegetables in the surroundings of food and nutrition public establishments

**DOI:** 10.1371/journal.pone.0294473

**Published:** 2023-11-30

**Authors:** Gabriela Gomes de Paiva, Rafael Moreira Claro, Bruna Vieira de Lima Costa

**Affiliations:** Department of Nutrition, Nursing School, Universidade Federal de Minas Gerais, Belo Horizonte, Minas Gerais, Brazil; Medical Research Council / Uganda Virus Research Institute & London School of Hygiene and Tropical Medicine Uganda Research Unit, UGANDA

## Abstract

**Background:**

Inequalities of access to healthy food reinforce the need to strengthen public policies on Food and Nutritional Security. In this context, Food and Nutrition Public Establishments, which are public spaces for distribution and commercialization of healthy foods, stand out. However, in middle-income countries there is little monitoring of the impacts of public policies on food environments, which are fundamental for strengthening these actions. Thus, the purpose of the study was to evaluate the availability and price of fruits and vegetables in areas with and without Food and Nutrition Public Establishments.

**Methodology:**

A cross-sectional study carried out in Belo Horizonte, Brazil. Audits were conducted in the retail food environment of a representative sample of Food and Nutrition Public Establishments (n = 10) and corresponding census sectors, without Food and Nutrition Public Establishments (n = 10). Information was collected on the availability and price of the most consumed fruits and vegetables in Belo Horizonte. The food retailers were grouped into fresh food retailers and mixed food retailers. The Chi-Square test was used to compare the availability of fruits and vegetables between areas. The Mann Whitney test was used to compare the prices of fruit and vegetables and the price differences between fresh food retailers and mixed food retailers between areas.

**Results:**

148 food retailers were audited, including 17 Food and Nutrition Public Establishments. In fresh food retailers, the availability of vegetables was higher in areas with Food and Nutrition Public Establishments (p = 0.024). In mixed food retailers there was low availability of fruit and vegetables in both areas (34.0% *vs*. 26.2%; p>0.05). The price difference between fresh food retailers and mixed food retailers differed significantly between areas (p<0.001). In general, fresh food retailers had lower prices than mixed food retailers only in areas with Food and Nutrition Public Establishments.

**Conclusions:**

The presence of Food and Nutrition Public Establishments was associated with the availability of vegetables, and with lower prices in fresh food retailers compared with mixed food retailers. Monitoring and understanding the impacts of public policies on the food environment are essential to register trends and provide relevant information to civil society and government authorities.

## Introduction

Fruits and vegetables (FV) are markers of a healthy diet [1, 2] and are therefore associated with a lower risk of morbidity and mortality [3, 4]. Despite its importance to health, FV consumption remains below recommended levels in most countries [5–8]. In many low and middle-income countries, purchasing the recommended levels would require a substantial portion of household income, making FV unaffordable [5–8]. As a matter of fact, FV represent the largest share of the costs to a healthy diet [9].

In Brazil, the increase in the social gap for adequate consumption of FV is concerning [8], and low-income families commit household income almost three times more than high-income families with the costs to a healthy diet [10]. Inequalities in FV access and consumption [5, 8] reinforce the need to improve access to healthy foods, which can be achieved through actions in the food environment [11–15].

The food environment is the interface between individuals and the food system [16, 17]. It encompasses dimensions of access to food that can influence people’s food acquisition and consumption [17]. The dimensions of access include availability, prices, affordability, accessibility and convenience of food and food retailers [17–20]. The importance of availability (i.e., types of food retailers and food available) and affordability (i.e., food price and purchasing power) stand out for FV consumption [5, 13, 20, 21].

Thus, the retail food environment wield significant influences on access [20], and barriers and facilitators for healthy food choices differ significantly among types of food retailers [22, 23]. For instance, in Brazil, fresh food is mostly acquired in open-air markets, greengroceries, butcheries, small producers, and street vendors [24, 25]. Although supermarkets have a larger market share with high participation in the acquisition of all types of food for Brazilians, it is important to highlight that most of the ultra-processed food comes from supermarkets [24].

Food and Nutrition Security public policies must be implemented through broad and synergistic actions that boost the distribution and commercialization of healthy foods [8]. In this context, the Food and Nutrition Public Establishments (FNPE), which are physical structures and public spaces designed to strengthening local circuits of food production, supply and distribution, stand out [26, 27]. FNPE that operate in the retail sector are food retailers focused on selling fresh foods at prices below those practiced in the market [26, 27]. The FNPE includes specialized fresh food public retailers, open-air markets, municipal markets, and places for direct purchase from regional producers [26, 27].

FNPE have some similarities with initiatives such as farmers markets, mobile markets, farm stands, and cost-offset community-supported agriculture, which are part of public Food and Nutrition Security policies in many countries in Latin America [11], Europe [28–32] and also in the United States [33–35]. In Brazil, FNPE are structural elements of the National Food and Nutritional Security Policy [23, 24]. They gained prominence in 2003 under the Zero Hunger strategy, launched by the federal government [23, 24]. Through bidding processes launched annually by the Ministry of Social Development, several states and municipalities joined the program [23, 24]. Currently, there are FNPE installed in 1652 medium and large Brazilian cities [23, 24]. Their structure varies between cities, in Belo Horizonte, they are implemented in areas with greater traffic of people, and there is a higher concentration of FNPE in areas of low socioeconomic vulnerability [36].

The FNPE are implemented to promote regular and permanent access to adequate and healthy food [23, 24]. However, there is little monitoring and evaluation of the impacts of public policies on food environments, which are fundamental for strengthening these actions [37]. It is necessary to consider that the application of FNPE takes place in complex spaces, which may affect availability and price variation in different ways in the various types of food retailers in the neighborhood [38–41]. Therefore, it is worth investigating the availability and price of food in FNPE and neighboring food retailers. Areas with FNPE are expected to have greater availability of FV sold at prices lower than those practiced in areas without FNPE. Thus, the study aimed to evaluate the availability and price of FV, according to the type of food retailers, in areas with and without FNPE, to better understand the food environment in the surroundings of FNPE.

## Methods

### Study design and sample

This is a cross-sectional study carried out in Belo Horizonte, Brazil. With 2.5 million inhabitants, it is the sixth most populous city in the country and the eighth in Latin America [42].

Between August and October 2019, store audits were carried out in the retail food environment of a representative sample of areas with EPSAN and corresponding areas (according to the region and socioeconomic status) with the absence of FNPE. The delimitation of the food environment was defined by a circular buffer with a radius of 500 meters around the FNPE, and in areas without FNPE, it was made around the centroid of the drawn census sector, enabled achieving a balance between test statistical power and sample size.

A complete list of FNPE and respective addresses was made available by the responsible municipal public body. All FNPEs selling food for home consumption and operating in May 2019 were considered eligible. A total of 116 public retail establishments were identified: specialized fresh food public retailers (i.e., places that sell mainly FV, with 16 items whose prices are set by the municipal administration) [43], open-air markets [i.e., mobile food retailers that operate on public roads, open on certain days of the week, selling fresh products] [44], municipal markets [large fixed structures, made up of small food retailers, which sell a variety of foods] [45] and direct purchase programs from local producers (i.e., marketing of fresh products directly from regional family farming producers) [46].

The study comprised two samples. First, a random sample was carried out by consecutive draws, stratified according to the nine administrative regions of Belo Horizonte and the socioeconomic vulnerability of the census tracts where the FNPE were located. The socioeconomic vulnerability was identified by the Health Vulnerability Index (HVI), a synthetic indicator constructed from socioeconomic data from the 2010 Demographic Census, from the Brazilian Institute of Geography and Statistics (IBGE as in Portuguese) [47]. A higher concentration of FNPE was identified in areas of low socioeconomic vulnerability, making it necessary to select two public establishments in the central region of Belo Horizonte, guaranteeing the proportionality of the sample. Thus, the minimum sample was defined as ten FNPE to represent the set of establishments with 95% confidence and an error margin of less than 3%. More information on the sampling process can be found elsewhere [48].

Second, the random sample of areas without FNPE were stratified by the administrative region of the city, consisting of 10 census tracts. Were considered eligible census tracts outside the region of influence of the FNPE, which was defined by a circular buffer with a radius of 1000 meters [45] around each FNPE. The census tracts should also have the same socioeconomic vulnerability identified in the area with the corresponding FNPE.

The areas delimited for investigation of the retail food environment were mapped and covered by the researchers to identify the food retail. A total of 253 food retailers were identified, of which 18 (7.1%) were located outside the units of analysis, and 87 (34.4%) refused to participate in the survey. All food retailer owners’ who participated in the study received information about the research and signed the Informed Consent Form. The study was approved by the Research Ethics Committee (CAAE 84707818.3.0000.5149).

## Retail food environment audit

The store audits of FNPE and food retailers was carried out in pairs, by a team of 10 researchers. The researchers were previously trained and monitored by field supervisors and the research coordinator.

Data collection used the “Food Store Observation Tool (ESAO-S)” and the “ESAO-S adapted to be used at open-air food markets”, tested and validated for Brazil [49]. Information was collected on availability (i.e., number of food retailers with available items) and price per kilo of the four FV (banana, orange, papaya, watermelon, pumpkin, tomato, carrot, and chayote) most consumed in Belo Horizonte, according to the 2008–2009 Household Budget Survey (HBS) [50]. Prices were assessed on the cheapest variety. Price comparisons were made for ’conventional’ products, excluding organic items.

### Classification of food retailers

The audited food retailers were grouped into: i) fresh food retailers (i.e., specialized fresh food public and private retailers, open-air markets, and direct purchase locations from local producers); and ii) Mixed food retailers (i.e., small warehouse, small market, grocery store, emporium, shack, and grocery stores; supermarkets and hypermarkets; bakeries, and convenience stores). The allocation of food retailers into the respective groups was based on the nature of the main products available, based on the grouping performed in a previous study of the Interministerial Chamber of Food and Nutritional Security [25].

### Statistical analyzes

The prevalence of the types of food retailers, and the availability of FV, were described using absolute and relative frequencies, for areas with and without FNPE. The currency of fresh food retailers and mixed food retailers was compared between regions with and without FNPE using Pearson’s chi-square test.

FV availability was compared between areas with and without FNPE, according to the types of food retailers; Pearson’s chi-square test was used. FV availability was not juxtaposed between fresh food retailers and mixed food retailers, as they were classified according to the nature of the main products available.

Means, minimum and maximum of FV prices in fresh food retailers and in mixed food retailers, according to their location in areas with and without FNPE, were used for descriptive purposes. The prices of FV were compared between the areas with and without FNPE, according to the types of food retailers, using the non-parametric Mann-Whitney test.

To compare the relationship between the price of FV in fresh food retailers and the price in mixed food retailers (price difference) between areas with and without FNPE, the price difference among each FV in fresh food retailers and the price in mixed food retailers, was calculated in two ways: i) difference between the average price in fresh food retailers, and the average price in mixed food retailers, calculated for areas with and without FNPE; and ii) price differences between fresh food retailers and mixed food retailers within each of the areas (with and without FNPE). The price difference between fresh food retailers and mixed food retailers was compared between areas. The difference between average prices (i) was used for descriptive purposes, and price differences between fresh food retailers and paired mixed food retailers (ii) were compared between areas with and without FNPE, using the non-parametric Mann-Whitney test. The non-parametric Mann-Whitney test was applied to compare continuous variables to avoid distribution assumptions and be more conservative concerning p values.

Statistical analyzes were conducted using the Stata version 14.2 statistical package. Values of p<0.05 were considered significant.

## Results

A total of ten areas with FNPE were included in the study, containing specialized fresh food public retailers (n = 2), street markets (n = 4), municipal markets (n = 2), and direct purchase programs from local producers (n = 2). Within the municipal markets, nine stores were investigated, considering the coexistence of different types of food retailers with independent commercialization. Thus, 17 FNPE units were audited. In total, 148 food retailers were audited, including the FNPE units.

Fresh food retailers were less prevalent than mixed food retailers, and there was no difference in the prevalence of types of retailers between areas (p>0.05) (Table 1).

**Table 1:** Types of food retailers according to areas. Belo Horizonte, Brazil. 2019.

Type of food retailer	Total	Areas without FNPE^1^	Areas with FNPE^1^
	n = 148	n = 76	n = 72
	% (n)	% (n)	% (n)
Fresh	39.9 (59)	38.2 (29)	41.7 (30)
Mixed	60.1 (89)	61.8 (47)	58.3 (42)

^1^Food and Nutrition Public Establishments

Regarding the availability of FV, in mixed food retailers there was low and similar availability between areas (34.0% vs. 26.2%; p>0.05). The availability of vegetables was higher in fresh food retailers in areas with FNPE when compared to fresh food retailers in areas without FNPE (100% vs. 82.8%; p = 0.024) (Table 2). Availability was tested for each FV investigated, agreeing with the results of total values.

**Table 2:** Availability of fruits and vegetables according to areas. Belo Horizonte, Brazil, 2019.

	Type of food retailer	Areas without FNPE^1^	Areas with FNPE^1^	χ^2^
		% (n^2^)	% (n^2^)	p-value
***Fruits***	Fresh	82.8 (24)	93.3 (28)	0.209
	Mixed	34.0 (16)	26.2(11)	0.421
***Vegetables***	Fresh	82.8 (24)	100.0 (30)	**0.024**
	Mixed	34.0 (16)	28.6 (12)	0.579

^1^ Food and Nutrition Public Establishments. ^2^ Food retailers that had availability of at least one variety of the investigated fruits and vegetables.

In general, similar values were observed when comparing the prices of the same food in the same type of food retailer–fresh food or mixed food–in areas with and without FNPE; in mixed food retailers, one of the items had lower prices in areas without FNPE (R$2.90 vs. R$3.85; p<0.05) (Table 3).

**Table 3:** Price of fruits and vegetables and price differentials between food retailers according to areas. Belo Horizonte, Brazil, 2019.

		Areas without FNPE^1^	Areas with FNPE^1^	Mann Whitney
*Fruits*		Mean (Min-Max^2^)	Mean (Min-Max^2^)	p-valor
Banana	Fresh food retailers (a)	R$3.35 (1.99–6.90)	R$3.38 (1.99–4.99)	0.1855
	Mixed food retailers (b)	R$2.90 (1.96–4.49)	R$3.85 (2.98–4.99)	**0.0156**
	Price difference^3^ (a—b)	+ R$0.45	-R$0.55	**0.0000**
Orange	Fresh food retailers (a)	R$2.40 (1.40–3.90)	R$2.37 (0.98–4.99)	0.9455
	Mixed food retailers (b)	R$1.96 (0.99–3.49)	R$2.49 (1.29–44.49)	0.2497
	Price difference^3^ (a—b)	+ R$0.44	-R$0.12	**0.0000**
Papaya	Fresh food retailers (a)	R$4.21 (1.99–8.98)	R$4.28 (1.48–6.99)	0.6744
	Mixed food retailers (b)	R$4.28 (1.99–9.90)	R$4.91 (2.98–6.99)	0.1456
	Price difference^3^ (a—b)	- R$0.07	- R$0.63	**0.0009**
Watermelon	Fresh food retailers (a)	R$1.90 (0.99–3.50)	R$2.14 (0.98–3.98)	0.0834
	Mixed food retailers (b)	R$2.46 (0.99–5.99)	R$1.82 (1.39–2.49)	0.6422
	Price difference^3^ (a—b)	-R$0.56	+ R$0.32	**0.0000**
***Vegetables***				
Pumpkin	Fresh food retailers (a)	R$2.32 (1.69–3.99)	R$2.29 (0.98–4.98)	0.4952
	Mixed food retailers (b)	R$1.94 (0.98–2.49)	R$2.36 (1.28–2.99)	0.0619
	Price difference^3^ (a—b)	+ R$0.38	- R$0.16	**0.0000**
Carrot	Fresh food retailers (a)	R$3.28 (1.99–8.00)	R$3.23 (1.48–6.98)	0.8392
	Mixed food retailers (b)	R$3.23 (1.38–6.20)	R$3.24 (1.49–5.49)	0.9345
	Price difference^3^ (a—b)	+ R$0.05	- R$0.01	0.1970
Tomato	Fresh food retailers (a)	R$3.66 (1.49–7.99)	R$3.42 (1.49-6-99)	0.8646
	Mixed food retailers (b)	R$3.65 (1.29–11.79)	R$4.15 (1.49–12.99)	0.8625
	Price difference^3^ (a—b)	+ R$0.01	- R$0.73	0.0727
Chayote	Fresh food retailers (a)	R$3.29 (1.78–4.99)	R$3.09 (1.98–4.98)	0.2946
	Mixed food retailers (b)	R$3.05 (0.99–4.99)	R$3.22 (2.29–3.99)	0.8140
	Price difference^3^ (a—b)	+ R$0.24	- R$0.13	**0.0008**

^1^Food and Nutrition Public Establishments.

^2^Minimum and maximum prices.

^3^The average price in fresh food retailers subtracted from the average price in mixed food retailers. p-values <0.05 are in bold.

However, the price difference between fresh food and mixed food retailers varied significantly between areas with and without FNPE (p<0.001 for six of the eight foods investigated). In general, price differences comparisons show that fresh food retailers are cheaper than mixed ones in areas with FNPE. On the other hand, in areas without FNPE, mixed retailers apparently are cheaper than fresh food retailers. For example, in areas with FNPE, the average banana price at fresh food retailers subtracted from the average banana price at mixed food retailers results in a difference of -R$0.55, (i.e. 16.3% lower at fresh food retailers). But, in areas without FNPE, the subtraction of the average banana price at fresh food retailers subtracted from the average banana price at mixed food retailers is R$0.45 (i.e. 13.4% higher at fresh food retailers) (Table 3).

## Discussion

Through store audits of a sample of 148 food retailers in areas with and without FNPE, it was possible to make progress in measuring the availability and price of FV in areas where a public policy on Food and Nutrition Security is operating. Fresh food retailers were less prevalent than mixed food retailers and were in similar proportions between areas with and without FNPE. In fresh food retailers, the availability of vegetables was higher in areas with FNPE. In mixed food retails, the availability of FV did not vary according to the areas, remaining low in both. In fresh food retailers, similar prices were observed in regions with and without FNPE. In mixed food retailers, one of the investigated items had higher prices in areas with FNPE. In general, price differences comparisons show that fresh food retailers are cheaper than mixed ones in areas with FNPE. On the other hand, in areas without FNPE, mixed retailers apparently are cheaper than fresh food retailers.

Most audited food retailers were the mixed type, reinforcing the hypothesis of similar distribution of establishment types in the country [25] and also in different urban centers [22, 51, 52]. However, our study adds new information by observing such distribution also concerning the presence of FNPE. There was no difference between the prevalence of these food retailers (fresh and mixed) according to the areas with and without FNPE, suggesting that the presence of the FNPE did not attract (or repel) food retailers in their surroundings. Environmental changes that drive responses to local food systems are complex and dynamic [53]. In addition to demand, the choice of a location for installing a new food retailer is based on strategies aimed at maximizing profits, to ensure the economic viability of the point of sale [54, 55]. In this logic, food retailers prioritize the richest areas of cities [54, 56–60]. In general, FV consumption is more common among better educated and wealthier people [61], and regular FV consumption is lower among lower-income individuals living in neighborhoods with fewer fresh food retailers [61].

Although FNPE did not interfere with the prevalence of establishments in their surroundings, their presence was associated with the availability of vegetables. In several countries, initiatives similar to FNPE demonstrate effectiveness in increasing the availability of FV in the regions where they are implemented [28–30, 33, 38]. It is known that the increased availability of FV in food retail is positively associated with increased consumption of these foods [17].

About the price of FV, it was possible to observe that the price difference between fresh food and mixed food retailers varied significantly between areas with and without FNPE, apparently because mixed retailers in areas with FNPE charge higher prices than those observed in the mixed retailers in areas without FNPE, as seem for one of the investigated items. Although our study is not able to present explanations for this scenario, two hypotheses must be considered: i) the presence of FNPE influences the type of food offered in the mixed retailers around them, causing them to focus on products of higher quality and higher income customers, who would be willing to pay higher values for convenience aspects [62]; and ii) the FNPEs are located in areas with a large natural flow of customers, concentrating in regions of low social vulnerability [36], a condition that also attracts food retailers more intensely [54, 56–60], precisely because of the possibility of profit maximization and implementation of higher prices [54, 62].

Large mixed food retailers, such as supermarkets and hypermarkets, have ample negotiating power [63]. In the last two decades, there has been a greater focus on agriculture and food production to supply these mixed food retailers [11], which gives them an advantage in commercial negotiation and the possibility of greater price control in the markets where they are installed [54]. However, in these food retailers, there is greater availability and dominance of ultra-processed foods [22–24, 52], which exposes consumers to a simultaneous offer of healthy and unhealthy foods. This availability and promotion of healthy and unhealthy foods in the same place seem to have ambiguous impacts on consumer eating behaviors, leading to ’matched’ purchases and, consequently, the acquisition and consumption of unplanned foods, which are commonly ultra-processed [61, 62]. Strategies present in mixed food retailers, such as greater availability, advertising, product promotion (tasting), price promotion, and physical modifications (such as consumer circulation through all sections and store; arrangement in islands or queues at checkouts or on shelves at eye level), encourage the purchase of ultra-processed foods [22, 24, 52, 64].

In general, price differences comparisons show that fresh food retailers are cheaper than mixed ones in areas with FNPE. On the other hand, in areas without FNPE, mixed retailers apparently are cheaper than fresh food retailers. It is known that food prices influence the choice of purchasing place [20] and the tendency to buy in fresh food retailers is more significant when these places have lower prices than other food retailers [65]. Increasing economic access to fresh food retailers is a crucial factor for the efficiency of intervention policies in the food environment [34]. Thus, the scenario observed in areas with FNPE could contribute to an increase in the propensity of choosing fresh food retailers as shopping places for consumers. The lowest price in fresh food retailers could be associated with the presence of FNPE. There are reports in the literature of reductions of up to 8% in food prices in food retailers adjacent to a retailer that charged FV prices lower than the local average [65]. However, it was not possible to find a difference between the prices charged in fresh food retailers in areas with FNPE compared to those in areas without FNPE. Naturally, only the larger FNPE has the actual capacity to interfere in the commercial structure of their surroundings, and given the great spatial variability of food prices, the identification of differences of small expressive magnitude (less than 20%) is conditioned to the use from samples with a much higher number of establishments than that of the present study.

In general terms, this study suggests that the food environment in areas with FNPE can lean toward healthy eating habits, by improving the availability of vegetables. Also, only in areas with FNPE, fresh food retailers presented lower price values than those observed in mixed food retailers, which can lead consumers to choose these food retailers. The price difference between fresh food retailers and mixed food retailers is not necessarily attributed to the presence of the FNPE and could be related to the socioeconomic status of the areas where these FNPE are mostly implemented. Broadly speaking, Food and Nutrition Security public policies aimed at making fresh food retailers more accessible and affordable could have wide benefits in FV consumption. Monitoring public policies in this field is essential for strengthening and expanding these actions in different contexts.

Some limitations of the study need to be pointed out. Data regarding the availability and price of FV were assessed simultaneously in a cross-sectional study. Thus, temporal variability in food availability and price may not have been measured. However, Brazil has a tropical climate, where the most consumed FV is produced all year round. And the availability of these foods tends to be consistent over two weeks, with a single observation reflecting commercial activities at the selected food retailers [66]. Another limitation related to food retailers that refused to participate in the study (34.4%), of these, 31% were fresh food retailers, and 69% were mixed food retailers, so the availability, and prices can be different. However, the distribution of the types of refusal food retailers did not differ from the distribution of the analyzed food retailers (p>0.01). Additionally, the circular buffers, drawn to delimit the investigation areas, establish boundaries of the food environment, which can be larger or smaller [67], but which are arbitrarily defined by the researcher, based on the literature [51, 61, 68].

Finally, the study design does not allow inferring a causal relationship between the presence of FNPE and the differences found between the areas. It is significant to consider the need for different approaches and designs, such as those used in this study, to try to clarify complex issues in environmental studies and to move forward [69]. In addition, monitoring and understanding the impacts of public intervention policies on the food environment are essential to register trends and provide relevant information to civil society and government authorities, however, the employment of traditional empirical methods to test the effectiveness of these strategies remains challenging [34].

## Conclusion

There was a higher prevalence of mixed food retailers, with no difference between areas with and without FNPE. The presence of FNPE was associated with the availability of vegetables. Overall, FV prices were similar in areas with and without FNPE. The price difference between fresh food retailers and mixed food retailers varied significantly between areas; in general, fresh food retailers presented lower price values than those observed in mixed food retailers, only in areas with FNPE. Adopting Food and Nutrition Security public policies can help to decrease the global burden of nutrition-related chronic diseases and inequities in health and access to healthy foods, and the application of relevant policies requires political leadership, broad social dialogue, and scientific information, as pointed out in the study. We used validated tools for auditing the retail food environment, aimed at monitoring public policies for physical and economic access to healthy foods; the importance of studies such as this one is highlighted, especially in low- and middle-income countries.

## Supporting information

S1 File (PDF)

S2 File (PDF)

## List of abbreviations

FV: Fruit and vegetables
FNPE: Food and Nutrition Public Establishments
HVI: Health Vulnerability Index
ESAO-S: Food Store Observation Tool

## References

[1] World Health Organization (WHO). “Best buys” and other recommended interventions for the prevention and control of noncommunicable diseases. World Heal Organ [Internet]. 2017;17(9]:28. Available at: http://apps.who.int/iris/bitstream/10665/259232/1/WHO-NMH-NVI-17.9-eng.pdf?ua=1

[2] Ministério da Saúde. Guia Alimentar para a População Brasileira. Secretaria de Atenção Primária à Saúde Departamento de Atenção Básica. 2014. 156 p. https://bvsms.saude.gov.br/bvs/publicacoes/guia_alimentar_populacao_brasileira_2ed.pdf

[3] Afshin A, Sur PJ, Fay KA, Cornaby L, Ferrara G, Salama JS, et al. Health effects of dietary risks in 195 countries, 1990–2017: a systematic analysis for the Global Burden of Disease Study 2017. Lancet. 2019;393(10184]:1958–72. 10.1016/S0140-6736(19)30041-8 30954305 PMC6899507

[4] Miller V, Mente A, Dehghan M, Rangarajan S, Zhang X, Swaminathan S, et al. Fruit, vegetable, and legume intake, and cardiovascular disease and deaths in 18 countries (PURE): a prospective cohort study. Lancet. 2017;390(10107):2037–49. doi: 10.1016/S0140-6736(17)32253-5 28864331

[5] Miller V, Yusuf S, Chow CK, Dehghan M, Corsi DJ, Lock K, et al. Availability, affordability, and consumption of fruits and vegetables in 18 countries across income levels: findings from the Prospective Urban Rural Epidemiology (PURE] study. Lancet Glob Heal. 2016;4(10]:e695–703. doi: 10.1016/S2214-109X(16)30186-3 27567348

[6] Oliveira N, Santin F, Paraizo TR, Sampaio JP, Moura-Nunes N, Canella DS. Baixa variedade na disponibilidade domiciliar de frutas e hortaliças no Brasil: dados das POF 2008–2009 e 2017–2018. Cien Saude Colet [Internet]. 26 de novembro de 2021;26(11]:5805–16. http://www.scielo.br/scielo.php?script=sci_arttext&pid=S1413-81232021001105805&tlng=pt 34852110 10.1590/1413-812320212611.25862020

[7] Kalmpourtzidou A, Eilander A, Talsma EF. Global vegetable intake and supply compared to recommendations: A systematic review. Nutrients. 2020;12(6]:22–9. doi: 10.3390/nu12061558 32471188 PMC7352906

[8] Crepaldi BVC, Okada LM, Rauber F, Levy RB, Azeredo CMH. Social inequality in food consumption between 2008 and 2019 in Brazil. Public Health Nutr. 2022;25(2]:214–24. doi: 10.1017/S1368980021002950 34407905 PMC8883783

[9] Hirvonen K, Bai Y, Headey D, Masters WA. Affordability of the EAT-Lancet reference diet: a global analysis. Lancet Glob Health. 2020 Jan;8(1):e59–e66. doi: 10.1016/S2214-109X(19)30447-4 Epub 2019 Nov 7. Erratum in: Lancet Glob Health. 2020 Dec;8(12):e1472. ; PMCID: PMC7024996. 31708415 PMC7024996

[10] Maia EG, Passos CM dos, Granado FS, Levy RB, Claro RM. Replacing ultra-processed foods with fresh foods to meet the dietary recomendations: a matter of cost?. Cad Saúde Pública [Internet]. 2021;37:e00107220. doi: 10.1590/0102-311X00107220 35019047

[11] FAO, FIDA, OPS, WFP y UNICEF. 2020. Panorama de la seguridad alimentaria y nutrición en América Latina y el Caribe 2020. Santiago de Chile. 10.4060/cb2242es

[12] HLPE. Food Security and Nutrition: Building a Global Narrative towards 2030. High Lev Panel Expert [Internet]. 2020;112. Available at: http://www.fao.org/3/ca9731en/ca9731en.pdf

[13] Ziso D, Chun OK, Puglisi MJ. Increasing Access to Healthy Foods through Improving Food Environment: A Review of Mixed Methods Intervention Studies with Residents of Low-Income Communities. Nutrients. 2022;14(11]. doi: 10.3390/nu14112278 35684077 PMC9182982

[14] Story M, Kaphingst KM, Robinson-O’Brien R, Glanz K. Creating healthy food and eating environments: Policy and environmental approaches. Annu Rev Public Health. 2008;29:253–72. doi: 10.1146/annurev.publhealth.29.020907.090926 . 18031223

[15] Drewnowski A, Monterrosa EC, de Pee S, Frongillo EA, Vandevijvere S. Shaping Physical, Economic, and Policy Components of the Food Environment to Create Sustainable Healthy Diets. Food Nutr Bull. 2020 Dec;41(2_suppl):74S–86S. doi: 10.1177/0379572120945904 . 33356590

[16] Downs SM, Ahmed S, Fanzo J, Herforth A. Food Environment Typology: Advancing an Expanded Definition, Framework, and Methodological Approach for Improved Characterization of Wild, Cultivated, and Built Food Environments toward Sustainable Diets. Foods. 2020 Apr 22;9(4):532. doi: 10.3390/foods9040532 ; PMCID: PMC7230632. 32331424 PMC7230632

[17] Turner C, Aggarwal A, Walls H, Herforth A, Drewnowski A, Coates J, et al. Concepts and critical perspectives for food environment research: A global framework with implications for action in low- and middle-income countries. Glob Food Sec [Internet]. 2018;18:93–101. doi: 10.1016/j.gfs.2018.08.003

[18] Clary C, Matthews SA, Kestens Y. Between exposure, access and use: Reconsidering foodscape influences on dietary behaviours. Health Place. 2017 Mar;44:1–7. doi: 10.1016/j.healthplace.2016.12.005 Epub 2017 Jan 11. . 28088114

[19] Caspi CE, Sorensen G, Subramanian S V., Kawachi I. The local food environment and diet: A systematic review. Heal Place [Internet]. setembro de 2012;18(5]:1172–87. Available at: 10.1016/j.healthplace.2012.05.006 PMC3684395 22717379

[20] Turner G, Green R, Alae-Carew C, Dangour AD. The association of dimensions of fruit and vegetable access in the retail food environment with consumption; a systematic review. Glob Food Sec [Internet]. junho de 2021;29:100528. Available at: doi: 10.1016/j.gfs.2021.100528 34164256 PMC8202327

[21] Slapø H, Schjøll A, Strømgren B, Sandaker I, Lekhal S. Efficiency of In-Store Interventions to Impact Customers to Purchase Healthier Food and Beverage Products in Real-Life Grocery Stores: A Systematic Review and Meta-Analysis. Foods. 2021 Apr 22;10(5):922. doi: 10.3390/foods10050922 ; PMCID: PMC8146080. 33922185 PMC8146080

[22] Borges CA, Gabe KT, Canella DS, Jaime PC. Characterization of barriers and facilitators for adequate and healthy eating in the consumer’s food environment. Cad Saude Publica. 2022 Feb 23;37 Suppl 1(Suppl 1):e00157020. English, Portuguese. doi: 10.1590/0102-311X00157020 . 35239773

[23] Machado PP, Claro RM, Canella DS, Sarti FM, Levy RB. Price and convenience: The influence of supermarkets on consumption of ultra-processed foods and beverages in Brazil. Appetite. 2017 Sep 1;116:381–388. doi: 10.1016/j.appet.2017.05.027 Epub 2017 May 17. . 28526478

[24] Machado PP, Claro RM, Martins APB, Costa JC, Levy RB. Is food store type associated with the consumption of ultra-processed food and drink products in Brazil? Public Health Nutr. 2018 Jan;21(1):201–209. doi: 10.1017/S1368980017001410 Epub 2017 Jul 31. ; PMCID: PMC10260784. 28756782 PMC10260784

[25] Brasil. Mapeamento dos Desertos Alimentares no Brasil. Câmara Interministerial de Segurança Alimentar e Nutricional. Ministério da Cidadania [Internet]. 2018;56. Available at: http://aplicacoes.mds.gov.br/sagirmps/noticias/arquivos/files/Estudo_tecnico_mapeamento_desertos_alimentares.pdf

[26] RedeSAN, FAURGS, UFRGS, MDS. Equipamentos Públicos de Segurança Alimentar e Nutricional [Internet]. 4o ed. Evangraf, organizador. News.Ge. Porto Alegre; 2011. 80 p. Available at: https://www.mds.gov.br/webarquivos/publicacao/seguranca_alimentar/equipamentospublicosSANpdf.pdf

[27] Brasil. Rede De Equipamentos Públicos De Alimentação E Nutrição: Resultados De Avaliações [Internet]. 2010. 164 p. Available at: https://fpabramo.org.br/acervosocial/

[28] von Philipsborn P, Geffert K, Klinger C, Hebestreit A, Stratil J, Rehfuess EA, et al. Nutrition policies in Germany: a systematic assessment with the Food Environment Policy Index. Public Health Nutr. 2022 Jun;25(6):1691–1700. doi: 10.1017/S1368980021004742 Epub 2021 Dec 9. ; PMCID: PMC9991688. 34881689 PMC9991688

[29] Adanacioglu H. Factors affecting the purchase behaviour of farmers’ markets consumers. PLoS One [Internet]. 2021;16(7 July]:1–17. Available at: doi: 10.1371/journal.pone.0255435 34329359 PMC8323914

[30] Klimek M, Bingen J, Freyer B. Metropolitan farmers markets in Minneapolis and Vienna: a values-based comparison. Agric Human Values. 2018;35(1]:83–97. https://link.springer.com/article/10.1007/s10460-017-9800-1

[31] Vecchio R. Italian and United States farmers’ markets: Similarities, differences and potential developments. J Food Prod Mark. 2011;17(2–3]:386–406. 10.1080/10454446.2011.548751

[32] Archer G.P., García Sánchez J., Vignali G. and Chaillot A. (2003), "Latent consumers’ attitude to farmers’ markets in North West England", British Food Journal, Vol. 105 No. 8, pp. 487–497. 10.1108/00070700310497264

[33] Janda KM, Ranjit N, Salvo D, Nielsen A, Akhavan N, Diaz M, et al. A Multi-Pronged Evaluation of a Healthy Food Access Initiative in Central Texas: Study Design, Methods, and Baseline Findings of the FRESH-Austin Evaluation Study. Int J Environ Res Public Health. 2021 Oct 15;18(20):10834. doi: 10.3390/ijerph182010834 ; PMCID: PMC8535966. 34682578 PMC8535966

[34] Salvo D, Lemoine P, Janda KM, Ranjit N, Nielsen A, van den Berg A. Exploring the Impact of Policies to Improve Geographic and Economic Access to Vegetables among Low-Income, Predominantly Latino Urban Residents: An Agent-Based Model. Nutrients. 2022 Feb 3;14(3):646. doi: 10.3390/nu14030646 ; PMCID: PMC8839639. 35277005 PMC8839639

[35] White MJ, Jilcott Pitts SB, McGuirt JT, Hanson KL, Morgan EH, Kolodinsky J, et al. The perceived influence of cost-offset community-supported agriculture on food access among low-income families. Public Health Nutr. 2018 Oct;21(15):2866–2874. doi: 10.1017/S1368980018001751 Epub 2018 Jul 11. ; PMCID: PMC10260852. 29991375 PMC10260852

[36] Lopes ACS, Menezes MC de, Araújo ML de. O ambiente alimentar e o acesso a frutas e hortaliças: “Uma metrópole em perspectiva”. Saude soc [Internet]. 2017 Jul;26(3):764–73. Available from: 10.1590/S0104-12902017168867

[37] Kumanyika S. INFORMAS (International Network for Food and Obesity/non-communicable diseases Research, Monitoring and Action Support): summary and future directions. Obes Rev. 2013 Oct;14 Suppl 1:157–64. doi: 10.1111/obr.12084 . 24074219

[38] Bekkers E, Brockmeier M, Francois J, Yang F. Local Food Prices and International Price Transmission. World Dev [Internet]. 2017;96:216–30. Available at: doi: http%3A//dx.doi.org/10.1016/j.worlddev.2017.03.008

[39] Allcott H, Diamond R, Dubé J-P, Handbury J, Rahkovsky I, Schnell M. Food Deserts and the Causes of Nutritional Inequality*. Q J Econ [Internet]. 1 de novembro de 2019;134(4]:1793–844. Available at: 10.1093/qje/qjz015

[40] Margaret E. Slade G.R.E.Q.A.M., Optimal Pricing with Costly Adjustment: Evidence from Retail-Grocery Prices, The Review of Economic Studies, Volume 65, Issue 1, January 1998, Pages 87–107, 10.1111/1467-937X.00036

[41] Leibtag E. The impact of big-box stores on retail food prices and the consumer price index. Price Dyn Behind Consum Food Purch. 2011;(January 2011):95–138. https://www.ers.usda.gov/webdocs/publications/45711/29160_err33_reportsummary.pdf?v=0

[42] Instituto Brasileiro de Geografia e Estatística (IBGE): Sinopse do Censo 2010 [Internet]. 261 p. Available at: https://biblioteca.ibge.gov.br/index.php/biblioteca-catalogo?view=detalhes&id=249230

[43] Prefeitura Municipal de Belo Horizonte (PBH). PBH Sacolões ABC.pdf [Internet]. 2019. Available at: https://prefeitura.pbh.gov.br/smasac/susan/comercializacao/sacolao-abastecer#:~:text=Ofertadeprodutosalimentícios%2Cespecialmente,deSegurançaAlimentareNutricional.

[44] Prefeitura Municipal de Belo Horizonte (PBH). PBH Feiras-livres.pdf [Internet]. 2022. Available at: https://prefeitura.pbh.gov.br/smasac/susan/comercializacao/feiras/feiras-livres

[45] Prefeitura Municipal de Belo Horizonte (PBH). PBH Mercado municipal.pdf [Internet]. 2022. Available at: https://prefeitura.pbh.gov.br/smasac/susan/comercializacao/mercados-municipais/mercado-distrital-do-cruzeiro

[46] Prefeitura Municipal de Belo Horizonte (PBH). PBH Direto da roça.pdf [Internet]. 2019. Available at: https://prefeitura.pbh.gov.br/smasac/direto-da-roca

[47] Pitchon A, Girodo A, Gomes C, Gomes D, Júnior F. Índice de Vulnerabilidade à Saúde. Índice Vulnerabilidade Da Saúde 2012 [Internet]. 2013;1–15. Available at: https://prefeitura.pbh.gov.br/sites/default/files/estrutura-de-governo/saude/2018/publicacaoes-da-vigilancia-em-saude/indice_vulnerabilidade2012.pdf

[48] Gomes Cordeiro N, Mendes LL, Jardim MZ, Claro RM, Pessoa MC, Granado FS, et al. Do Food and Nutrition Public Establishments Influence Availability to Healthy Food in Neighborhood? J Hunger Environ Nutr [Internet]. 8 de dezembro de 2022;1–18. Available at: 10.1080/19320248.2022.2155095

[49] Duran AC, Lock K, Latorre Mdo R, Jaime PC. Evaluating the use of in-store measures in retail food stores and restaurants in Brazil. Rev Saude Publica. 2015;49:80. doi: 10.1590/S0034-8910.2015049005420 Epub 2015 Oct 30. ; PMCID: PMC4617430. 26538101 PMC4617430

[50] Instituto Brasileiro de Geografia e Estatística (IBGE). Pesquisa de Orçamentos Familiares: 2008–2009. Análise do Consumo Alimentar Pessoal no Brasil [Internet]. 2011. 150 p. Available at: http://biblioteca.ibge.gov.br/visualizacao/livros/liv50063.pdf

[51] Curioni CC, Boclin KLS, Silveira IH, Canella DS, Castro IRR, Bezerra FF, et al. Neighborhood food environment and consumption of fruit and leafy vegetables: Pro-Saude Study, Brazil. Public Health [Internet]. 2020;182:7–12. doi: 10.1016/j.puhe.2020.01.004 32112980

[52] Serafim P, Borges CA, Cabral-Miranda W, Jaime PC. Ultra-Processed Food Availability and Sociodemographic Associated Factors in a Brazilian Municipality. Front Nutr. 2022 Apr 20;9:858089. doi: 10.3389/fnut.2022.858089 ; PMCID: PMC9067397. 35529462 PMC9067397

[53] Ghosh-Dastidar M, Hunter G, Collins RL, Zenk SN, Cummins S, Beckman R, et al. Does opening a supermarket in a food desert change the food environment? Heal Place [Internet]. 2017;46(June]:249–56. doi: http%3A//dx.doi.org/10.1016/j.healthplace.2017.06.002 28648926 10.1016/j.healthplace.2017.06.002 PMC5588682

[54] Gouri Suresh SS, Schauder SA. Income Segregation and Access to Healthy Food. Am J Prev Med [Internet]. 2020;59(2]:e31–8. 10.1016/j.amepre.2020.02.009 32418802

[55] Current J, Min H, Schilling D. Multiobjective analysis of facility location decisions. Eur J Oper Res [Internet]. 1990;49(3]:295–307. Available at: https://www.sciencedirect.com/science/article/pii/037722179090401V

[56] Costa BV de L, Oliveira CDL, Lopes ACS. Food environment of fruits and vegetables in the territory of the Health Academy Program. Cad Saúde Pública [Internet]. 2015Nov;31:159–69. Available from: doi: 10.1590/0102-311X00027114 26648371

[57] Duran AC, Diez Roux AV, Latorre Mdo R, Jaime PC. Neighborhood socioeconomic characteristics and differences in the availability of healthy food stores and restaurants in Sao Paulo, Brazil. Health Place. 2013 Sep;23:39–47. doi: 10.1016/j.healthplace.2013.05.001 Epub 2013 May 18. ; PMCID: PMC3758426. 23747923 PMC3758426

[58] Jaime PC, Duran AC, Sarti FM, Lock K. Investigating environmental determinants of diet, physical activity, and overweight among adults in Sao Paulo, Brazil. J Urban Health. 2011 Jun;88(3):567–81. doi: 10.1007/s11524-010-9537-2 ; PMCID: PMC3126927. 21327549 PMC3126927

[59] Leite MA, Assis MM, Carmo ASD, Costa BVL, Claro RM, Castro IR, et al. Is neighbourhood social deprivation in a Brazilian city associated with the availability, variety, quality and price of food in supermarkets? Public Health Nutr. 2019 Dec;22(18):3395–3404. doi: 10.1017/S1368980019002386 Epub 2019 Aug 29. ; PMCID: PMC10260548. 31462336 PMC10260548

[60] Pessoa MC, Mendes LL, Gomes CS, Martins PA, Velasquez-Melendez G. Food environment and fruit and vegetable intake in a urban population: A multilevel analysis. BMC Public Health [Internet]. 2015;15(1]:1–8. doi: http%3A//dx.doi.org/10.1186/s12889-015-2277-1 26437719 10.1186/s12889-015-2277-1 PMC4595198

[61] Duran AC, de Almeida SL, Latorre Mdo R, Jaime PC. The role of the local retail food environment in fruit, vegetable and sugar-sweetened beverage consumption in Brazil. Public Health Nutr. 2016 Apr;19(6):1093–102. doi: 10.1017/S1368980015001524 Epub 2015 Jun 9. ; PMCID: PMC10271044. 26054646 PMC10271044

[62] Faber B, Fally T. Firm Heterogeneity in Consumption Baskets: Evidence from Home and Store Scanner Data. Rev Econ Stud [Internet]. 1 de maio de 2022;89(3]:1420–59. 10.1093/restud/rdab061

[63] Taillie LS, Ng SW, Popkin BM. Global growth of "big box" stores and the potential impact on human health and nutrition. Nutr Rev. 2016 Feb;74(2):83–97. doi: 10.1093/nutrit/nuv062 Epub 2015 Dec 29. ; PMCID: PMC4892305. 26714934 PMC4892305

[64] Stanton RA. Food Retailers and Obesity. Curr Obes Rep. 2015 Mar;4(1):54–9. doi: 10.1007/s13679-014-0137-4 . 26627090

[65] McGuirt JT, Jilcott Pitts SB, Ward R, Crawford TW, Keyserling TC, Ammerman AS. Examining the Influence of price and accessibility on willingness to shop at farmers’ markets among low-income eastern North Carolina women. J Nutr Educ Behav. 2014 Jan;46(1):26–33. doi: 10.1016/j.jneb.2013.06.001 Epub 2013 Nov 5. ; PMCID: PMC3891513. 24201077 PMC3891513

[66] Zenk SN, Grigsby-Toussaint DS, Curry SJ, Berbaum M, Schneider L. Short-term temporal stability in observed retail food characteristics. J Nutr Educ Behav. 2010 Jan-Feb;42(1):26–32. doi: 10.1016/j.jneb.2009.01.005 ; PMCID: PMC2913966. 20129186 PMC2913966

[67] Seliske L, Pickett W, Rosu A, Janssen I. Identification of the appropriate boundary size to use when measuring the food retail environment surrounding schools. Int J Environ Res Public Health. 2012 Aug;9(8):2715–27. doi: 10.3390/ijerph9082715 Epub 2012 Jul 31. ; PMCID: PMC3447582. 23066392 PMC3447582

[68] Costa BVL, Menezes MC, Oliveira CDL, Mingoti SA, Jaime PC, Caiaffa WT, et al. Does access to healthy food vary according to socioeconomic status and to food store type? an ecologic study. BMC Public Health. 2019 Jun 18;19(1):775. doi: 10.1186/s12889-019-6975-y ; PMCID: PMC6582565. 31215435 PMC6582565

[69] Gomes CS, Silveira EA, Velasquez-Melendez G. Neighborhood environment is associated with unhealthy food intake in a Brazilian urban area. Appetite. 2022 May 1;172:105972. doi: 10.1016/j.appet.2022.105972 Epub 2022 Feb 15. . 35176434

