# Supplementary material for: Availability and price of fruits and vegetables in the surroundings of food and nutrition public establishments
Source: PLoS One. 2023 Nov 30;18(11):e0294473. doi: 10.1371/journal.pone.0294473 (PMC10688891; doi:10.1371/journal.pone.0294473)
Supplement: S1 File — (PDF) [file pone.0294473.s001.pdf]

**TERMS OF FREE AND CLEAR CONSENT**

Dear participant,

In accordance with Resolution nº 196/96 of the National Health Council and as required by the Research Ethics Committee, I would like to introduce myself to you and invite you to participate in the research "Food Environment in the Territory of Food and Nutritional Safety Equipment in the City of Belo Horizonte". The research aims to evaluate the food environment in the territory of public facilities for food and nutritional security in Belo Horizonte.

For this study, a direct on-site observation of your commercial establishment will be carried out to assess the quality, price, advertising, availability and variety of food offered.

You are free not to participate in the research and this will not cause you any harm. In addition, you will have no expenses and no financial benefits.

The risk, in this study, is restricted to the secrecy of identification and information collected in its establishment. However, all care regarding secrecy will be carried out to preserve the identity of your establishment and information about it. In this way, the researchers undertake to take care of the information confidentially. For this, the documents filled in at your establishment after the end of the research will be kept confidential by the researcher in charge (Professor Bruna Vieira de Lima Costa) and if they are discarded at some point, the researcher undertakes to shred all the material maintaining the secrecy of the information collected.

The survey will provide you with the opportunity to contribute to the understanding of food marketing around public food and nutrition security facilities. It will be possible to verify the differences in the density, distribution and quality of the types of food establishments. This study will also collaborate with the prevention of obesity through public policies that seek to improve the supply of healthy foods.

You will receive a copy of this FORM OF FREE AND CLEAR CONSENT - TCLE and if there is any information you wish to receive, the contact telephone number is (0xx31 – 3409-8028 and 0xx31 - 34098038). If you are interested, you may also contact the Research Ethics Committee (COEP) of the Federal University of Minas Gerais by phone (0xx31-34094592).

Thank you in advance for your attention and collaboration.

I believe I was informed about what I read or what was read to me about the research "Food Environment in the Territory of Public Equipment for Food and Nutrition Security in the City of Belo Horizonte". It was clear to me what the objectives of the study are, and what measures will be collected, their risks and discomforts. I declare that I am aware that all information is confidential and that I have the guarantee of clarifying any doubts. I know that my participation will have no expenses or remuneration and that my rights are preserved. Thus, I voluntarily agree and consent to my participation in the study, and I may withdraw my consent at any time, before or during it, without any prejudice.

Name: ____________________________________________________

Signature ___________________________________________________

Date:___/___/______

I declare that I have voluntarily obtained Free and Informed Consent to participate in this study.

_______________________________________________

Bruna Vieira de Lima Costa – Research Coordinator

(Phone: 34098038)

Project coordinator: Prof. Dr. Bruna Vieira de Lima Costa

Federal University of Minas Gerais School of Nursing – UFMG

Nutrition Course - Department of Nutrition

Av. Alfredo Balena, 190 – 3rd. Floor – Room 314 - Bairro Santa Efigênia

CEP 30130-100 – (31) 3409-8038 – Belo Horizonte – MG

COEP UFMG

Av. Pres. Antônio Carlos, 6627 – Administrative Unit II - 2nd floor – Room 2005

Zip Code: 31270-901 – BH – MG

Fax: (31) 34094592 – email: coep@prpq.ufmg.br
