# Supplementary material for: Availability and price of fruits and vegetables in the surroundings of food and nutrition public establishments
Source: PLoS One. 2023 Nov 30;18(11):e0294473. doi: 10.1371/journal.pone.0294473 (PMC10688891; doi:10.1371/journal.pone.0294473)
Supplement: S2 File — (PDF) [file pone.0294473.s002.pdf]

TERMO DE CONSENTIMENTO LIVRE E ESCLARECIDO

Caro participante,

De acordo com a Resolução nº 196/96 do Conselho Nacional de Saúde e conforme requisito do Comitê de Ética em Pesquisa, me apresento a você e venho convidar-lhe a participar da pesquisa "Ambiente Alimentar no Território dos Equipamentos de Segurança Alimentar e Nutricional da Cidade de Belo Horizonte". A pesquisa tem como objetivo de avaliar o ambiente alimentar no território dos equipamentos públicos de segurança alimentar e nutricional de Belo Horizonte.

Para este estudo será realizada uma observação direta *in loco* do seu estabelecimento comercial para avaliação da qualidade, preço, propaganda, disponibilidade e variedade dos alimentos ofertados.

Você tem liberdade em não participar da pesquisa e isso não lhe trará nenhum prejuízo. Além disso, você não terá nenhuma despesa e nenhum benefício financeiro.

O risco, neste estudo, restringe-se ao sigilo da identificação e às informações coletadas no seu estabelecimento. Contudo, todos os cuidados quanto ao sigilo serão realizados para preservar a identidade do seu estabelecimento e das informações sobre o mesmo. Desta forma, os pesquisadores assumem o compromisso de cuidar das informações de maneira sigilosa. Para isso, os documentos preenchidos em seu estabelecimento após o término da pesquisa serão guardados sob sigilo pela pesquisadora responsável (Profa. Bruna Vieira de Lima Costa) e caso sejam descartados em algum momento a pesquisadora se compromete a picotar todo o material mantendo o sigilo das informações coletadas.

A pesquisa irá lhe proporcionar a oportunidade de contribuir para compreensão da comercialização de alimentos ao redor dos equipamentos públicos de segurança alimentar e nutricional. Será possível verificar as diferenças na densidade, distribuição e qualidade dos tipos de estabelecimentos de alimentos. Este estudo também irá colaborar com a prevenção da obesidade por meio de políticas públicas que busquem melhorar a oferta de alimentos saudáveis.

O senhor(a) irá receber uma cópia deste TERMO DE CONSENTIMENTO LIVRE E ESCLARECIDO – TCLE e se houver alguma informação que deseje receber, o telefone de contato é (0xx31 – 3409-8028 e 0xx31 - 34098038). Caso seja de seu interesse também poderá entrar em contato com o Comitê de Ética em Pesquisa (COEP) da Universidade Federal de Minas Gerais pelo telefone (0xx31- 34094592).

Desde já agradeço sua atenção e colaboração.

Acredito ter sido informado a respeito do que li ou do que foi lido para mim sobre a pesquisa "Ambiente Alimentar no Território dos Equipamentos Públicos de Segurança Alimentar e Nutricional da Cidade de Belo Horizonte". Ficaram claros para mim quais são os objetivos do estudo, e quais medidas serão coletadas, seus riscos e desconfortos. Declaro ciente que todas as informações são confidenciais e que eu tenho a garantia de esclarecimento de qualquer dúvida. Sei que a minha participação não terá despesas, nem remuneração e que estão preservados os meus direitos. Assim, concordo voluntariamente e consinto na minha participação no estudo, sendo que poderei retirar meu consentimento a qualquer momento, antes ou durante o mesmo, sem quaisquer prejuízos.

Nome: ____

Assinatura ____

Data:___/___/______

Declaro que obtive de forma voluntária o **Consentimento Livre e Esclarecido** para participação neste estudo.

____

Bruna Vieira de Lima Costa – Coordenadora da Pesquisa
(Telefone: 34098038)

Coordenadora do projeto: Profa. Dra. Bruna Vieira de Lima Costa
Escola de Enfermagem da Universidade Federal de Minas Gerais – UFMG
Curso de Nutrição - Departamento de Nutrição
Av. Alfredo Balena, 190 – 3º. Andar – Sala 314 - Bairro Santa Efigênia
CEP 30130-100 – (31) 3409-8038 – Belo Horizonte – MG

COEP UFMG
Av. Pres. Antônio Carlos, 6627 – Unidade Administrativa II - 2° andar – Sala 2005
Cep: 31270-901 – BH – MG
Telefax: (31) 34094592 – e-mail: coep@prpq.ufmg.br
